# Medical Cannabis Use Reduces Opioid Prescriptions in Patients With Chronic Back Pain

**DOI:** 10.7759/cureus.21452

**Published:** 2022-01-20

**Authors:** Ari Greis, Bryan Renslo, Adrianne R Wilson-Poe, Conan Liu, Anjithaa Radakrishnan, Asif M Ilyas

**Affiliations:** 1 Department of Medical Cannabis, Rothman Orthopaedic Institute, Philadelphia, USA; 2 Department of Medical Cannabis, Sidney Kimmel Medical College at Thomas Jefferson University, Philadelphia, USA; 3 Rothman Orthopaedic Institute Foundation for Opioid Research & Education, Rothman Orthopaedic Institute, Philadelphia, USA; 4 Dow Neurobiology Labs, Legacy Research Institute, Portland, USA

**Keywords:** prescription opioid, opioids use, cannabis (marijuana), cannabis research, chronic low back pain (clbp), chronic pain management

## Abstract

Background

This study investigates whether the use of medical cannabis (MC) in patients with chronic back pain is associated with a decreased opioid prescription.

Methods

The study included 186 patients with chronic back pain who were certified for MC use. The average morphine milligram equivalent (MME)/day of opioid prescriptions filled within the six months prior to MC certification was compared to that of six months after. Pain and disability questionnaires were distributed at three, six, and nine months post-certification.

Results

Patients who started at less than 15 MME/day and patients who started at greater than 15 MME/day decreased from 15.1 to 11.0 (n = 186, p < 0.01), 3.5 to 2­­­.1 (n = 134, p < 0.01), and 44.9 to 33.9 (n = 52, p < 0.01), respectively. Pain and disability scores were improved at follow-up as well.

Conclusion

MC use reduces opioid prescription for patients with chronic back pain and improves pain and disability scores.

## Introduction

Opioid abuse is an ongoing problem, with opioid-related deaths nearly quadrupling between 1999 and 2015 in the United States [1]. Deaths related to prescription opioids peaked in 2017, had briefly declined, and now continue to increase [2]. Opioids are still routinely used for chronic pain, including nonspecific generalized chronic low back pain, as their use has shown small improvements in pain, physical functioning, and sleep quality [3,4]. The benefits of opioid use come at the cost of dose-dependent risks of substance abuse disorders, addiction, overdose, and death [5]. Alternate therapies for chronic pain are needed to mitigate these adverse outcomes and reduce societal problems related to opioid use.

Medical cannabis (MC) is an emerging therapeutic option for adult patients with chronic pain, but its efficacy is not fully confirmed. MC has been approved for the treatment of pain in over 30 states as of 2020, making it much more widely available [6]. MC has been shown to reduce chronic noncancer pain, although its efficacy is not well proven when compared to a control group [7]. The use of MC for neuropathic pain and multiple sclerosis-related spasticity has also been shown to be efficacious; however, these studies are limited by small sample sizes and the short duration of the study [8,9]. MC has been shown to be an effective treatment of orthopedic pain when compared to placebo, but this effectiveness has not yet been shown when compared to an active comparator [10]. Heterogeneity in the literature regarding efficacy, route, dosing, and safety of MC use for chronic pain makes it difficult to develop solid guidelines for how MC should be used. One study has shown that overuse of MC can paradoxically increase the intensity of chronic pain when used in high frequency [11]. Additionally, regular MC users have shown decreased neurocognitive function compared to nonusers in cognitive and behavioral laboratory testing, although these effects have not been observed in a clinical setting [12].

There are limited data to support MC as an effective replacement for opioids. However, population studies show that the legalization of MC has been associated with reduced mortality due to opioid overdose, reduced opioid-related hospitalizations, and decreased opioid prescription [13]. This study was undertaken to better understand the association between the certification of MC use and opioid utilization for the management of chronic musculoskeletal noncancer low back pain. The study hypothesis was that MC certification and use for chronic low back pain would result in decreased opioid prescriptions filled.

## Materials and methods

Patient outcome measures for this study were obtained by querying a prospectively collected cannabis data repository. Patients with a diagnosis of chronic musculoskeletal noncancer low back pain who were certified for MC between February 2018 and July 2019 were prospectively enrolled and included. Patients were included in the analysis if they had a diagnosis of the spine, were not indicated for surgical or injection treatment, and were actively consuming opioids. Exclusion criteria included patients with all non-spine diagnoses, having undergone surgery within six months before or after MC certification, and not consuming opioids at the time of MC certification.

Patient demographic information, visual analog scale (VAS) back pain score, numeric back pain intensity, numeric back pain frequency, VAS right leg pain score, VAS left leg pain score, numeric leg pain frequency, numeric leg pain intensity, and Oswestry Disability Index (ODI) scores were collected. VAS and numeric scales for pain, as well as the ODI, are commonly used metrics for the evaluation of lower back pain [14-16]. Scores were collected by outcome measure analysts not involved in the present study.

All physicians involved with MC certification previously attended a four-hour continuing medical education (CME) course and applied to the Pennsylvania Department of Health to become approved practitioners. MC was certified for patients who met the criteria by being a resident of Pennsylvania and suffering from one of the 23 state-approved medical conditions [17]. During the certification visit, the chemical constituents of MC, routes of delivery, optimal dosing parameters, and potential risks were reviewed with the patient. Patients were required to sign an informed consent form. For patients naïve to MC, it was recommended they start with low dosages of tetrahydrocannabinol (THC) to limit psychoactive side effects, oftentimes combined with cannabidiol (CBD). An oral route of delivery, often with a sublingual tincture, and/or topical cannabinoids were recommended over vaporization, while smoking was not endorsed. Once certified, patients could purchase an MC identification card from the Pennsylvania Department of Health and shop at state-approved dispensaries.

Data regarding filled prescriptions of all controlled substances were gathered using Pennsylvania’s Prescription Drug Monitoring Program (PDMP) system. PDMP is a state-run program that collects information on all filled prescriptions for controlled substances and can be accessed online by licensed providers. Data on opioid prescriptions that were filled within six months before and after MC certification were collected into a password-protected Microsoft Excel (Microsoft Corporation, Redmond, WA) database. Opioid use was calculated based on the average morphine milligram equivalents (MME) filled per day over the six months before and after MC certification. Patients were then stratified into greater than or less than 15 MME/day groups based on average MME per day prior to MC certification. A threshold of 20 MME/day has been used in the past based on data showing increased rates of overdose in patients taking over 20 MME/day [5,18]. The threshold of 15 MME/day was used for this study given that it represents a typical consumption of two to three opioid pills daily. Primary study outcome measures consisted of the change in opioid use within six months pre- and post-MC certification and use. Secondary study outcome measures included pain, daily function scores, and adverse effects. Data on adverse effects and route of MC administration were gathered during the three to six months follow-up visit and retrospectively collected.

Statistics were calculated within Microsoft Excel using a paired, two-tailed t-test for paired data, a two-tailed t-test with unequal variance for non-paired data, and a single-factor analysis of variance for multi-group comparison. An alpha threshold of alpha = 0.05 was applied for all unique significance tests.

## Results

**Figure 1:**
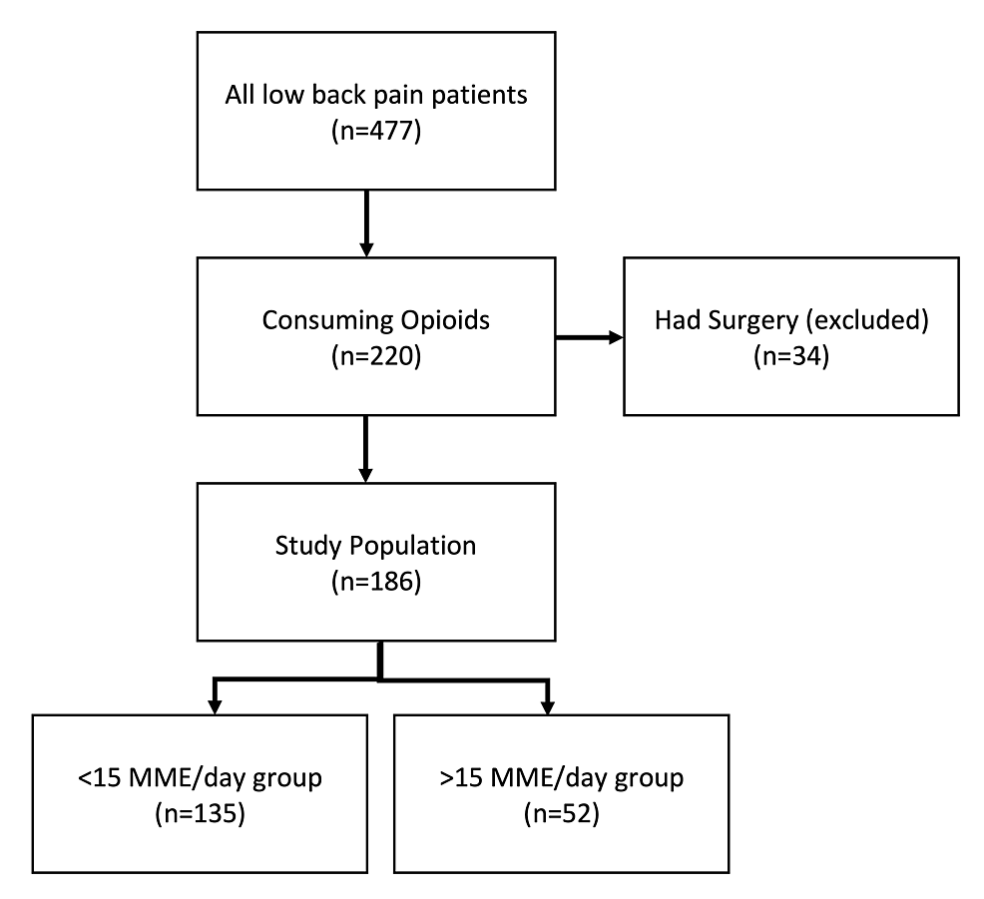
Number of patients who filled controlled substance prescriptions within six months before medical cannabis prescription. Of all patients with chronic low back pain (n = 477), 220 patients were actively consuming opioids. Patients who had surgery within six months of medical cannabis certification were removed (n = 34). Our study population included 186 patients. The study population was further divided into patients taking less than 15 morphine milligram equivalents per day (<15 MME/day, n = 135) prior to medical cannabis or more than 15 morphine milligram equivalents per day (>15 MME/day, n = 52).

A total of 632 patients were certified for MC for a musculoskeletal pain diagnosis between February 2018 and July 2019. Of those patients, 477 had a diagnosis related to chronic low back pain (Figure 1). A total of 186 patients met the study’s inclusion criteria, of which 87 (46.8%) were male and 99 (53.2%) were female. The average age at the date of MC certification was 64.0 years (range 33-90 years). The patients were then stratified into groups based on whether their opioid fill rates were less than 15 MME/day or greater than 15 MME/day (Figure 1). Patient diagnoses are listed in Table 1.

**Table 1:** Patient diagnoses. Radicular includes a diagnosis of radiculopathy, disc displacement, degenerative disc disease, pain syndrome, neuropathy, disc herniation, neuritis, and radiculitis. Degenerative includes a diagnosis of spinal stenosis, spondylosis, arthrodesis, spinal fusion, arthropathy, sacroiliitis, back pain (unspecified), and postlaminectomy syndrome. Deformity includes a diagnosis of spondylolisthesis and scoliosis.

Category	N
Radicular	88
Degenerative	81
Deformity	17

**Figure 2:**
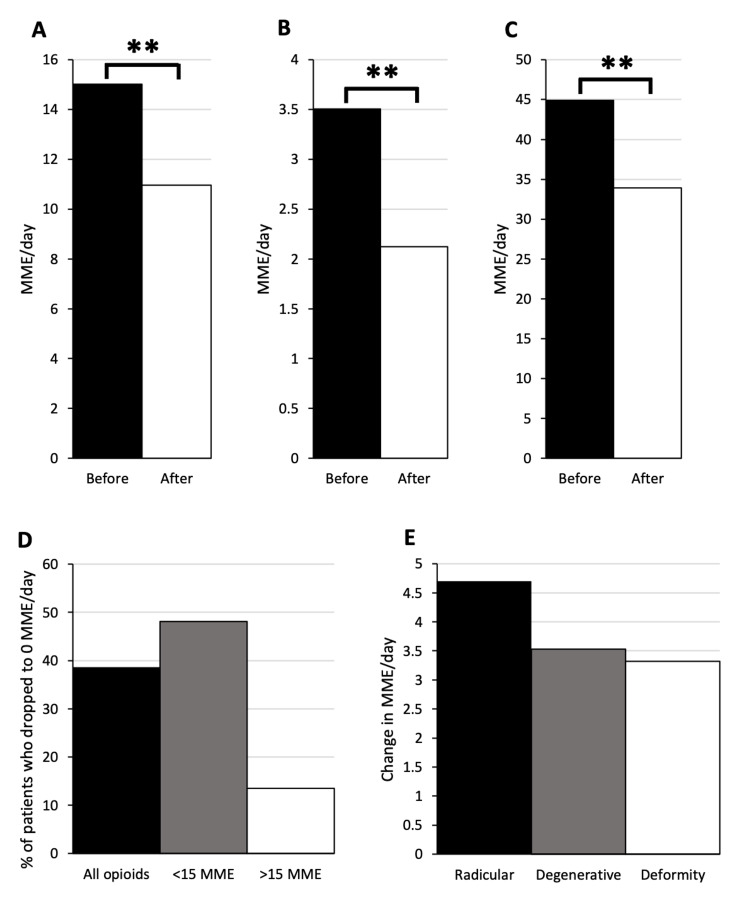
Opioid group shows decreased opioid prescriptions filled post medical cannabis prescription. All patients on opioids (A) showed a drop in morphine milligram equivalents per day (MME/day) from 15.1 to 11.0 (n = 186, **p < 0.01). Patients below 15 MME/day (B) showed a drop from 3.5 to 2.1 (n = 134, **p < 0.01). Patients above 15 MME/day pre-medical cannabis (C) showed a drop from 44.9 to 33.9 (n = 52, **p < 0.01). All patients on opioids, patients below 15 MME/day, and patients above 15 MME/day had a 38.7%, 48.5%, and 13.5%, drop to 0 rate, respectively (D). There was no significant difference in change in MME/day between patients with radicular (n = 88), degenerative (n = 81), or deformity (n = 17) etiology of pain (4.7 vs. 3.5 vs. 3.3, p = 0.88) (E).

Average MME/day dropped from 15.1 to 11.0 (n = 186, p < 0.01) between six months pre-MC to six months post-MC (Figure 2A). The percentage of patients who dropped to 0 MME/day was 38.7% (Figure 2D). The average drop in MME/day was 27.0%. For patients below 15 MME/day pre-MC, average MME/day dropped from 3.5 to 2.1 (n = 134, p < 0.01; Figure 2B). The percentage of patients who dropped to 0 MME/day was 48.5% (Figure 2D). The average change in MME/day was 39.7%. For patients above 15 MME/day pre-MC, average MME/day dropped from 44.9 to 33.9 (n = 52, p < 0.01; Figure 2C). The percentage of patients who dropped to 0 MME/day was 13.5% (Figure 2D). The average change in MME/day was 24.5%. Average drop in MME/day was compared between patients with radicular (n = 88), degenerative (n = 81), or deformity (n = 17) etiology of pain. There was no significant difference between the groups (4.7 vs. 3.5 vs. 3.3, p = 0.88; Figure 2E).

**Figure 3:**
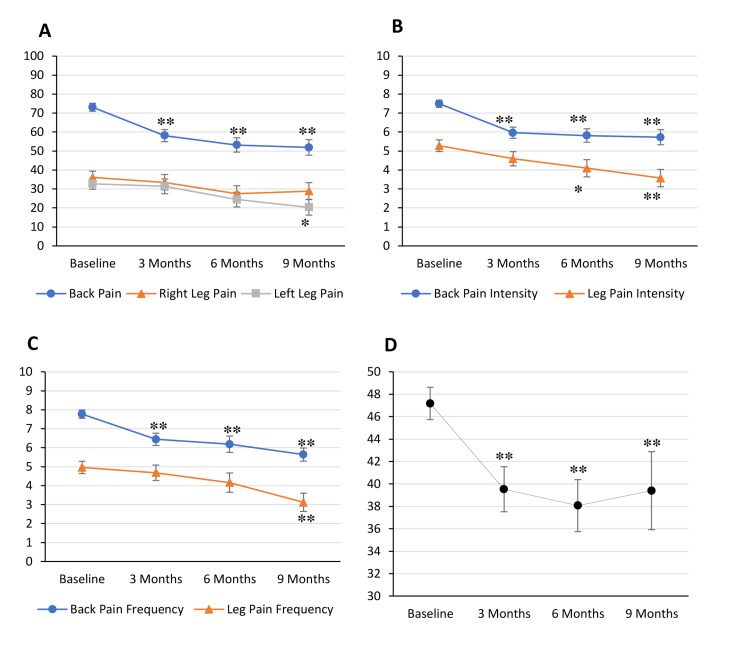
Pain and Oswestry Disability Index (ODI) scores decrease from baseline following medical cannabis certification. Average patient scores were measured at baseline (n = 100) and at three (n = 60), six (n = 44), and nine months (n = 33) following medical cannabis (MC) certification. Visual analog scale (VAS) back pain score decreased significantly from 73.1 at baseline to 58.1 (**p < 0.01), 53.2 (**p < 0.01), and 51.9 (**p < 0.01) at three, six, and nine months, respectively (A). VAS right leg pain score decreased insignificantly from 36.1 to 33.5, 27.5, and 28.9 (A). VAS left leg pain score decreased insignificantly from 32.7 to 31.4 at three months and 24.4 at six months and decreased significantly to 20.3 (*p < 0.05) at nine months (A). Back pain intensity score decreased significantly from 7.5 to 6.0 (**p < 0.01), 5.8 (**p < 0.01), and 5.7 (**p < 0.01, B). Leg pain intensity score decreased insignificantly from 5.3 to 4.6 at three months and decreased significantly to 4.1 (*p < 0.05) at six months and 3.6 (**p < 0.01) at nine months (B). Back pain frequency decreased significantly from 7.8 to 6.4 (**p < 0.01), 6.2 (**p < 0.01), and 5.6 (**p < 0.01) (C). Leg pain frequency decreased insignificantly from 5.0 to 4.7 at three months and 4.2 at six months and decreased significantly to 3.1 (**p < 0.01) at nine months (C). ODI score decreased significantly from 47.2 to 39.5 (**p < 0.01), 38.1 (**p < 0.01), and 39.4 (**p < 0.01) (D). Error bars represent standard error.

Pain scores and ODI scores were measured at baseline (n = 100) and three (n = 60), six (n = 44), and nine months (n = 33) post-MC certification. Compared to baseline, VAS back pain score decreased significantly from 73.1 to 58.1 (p < 0.01), 53.2 (p < 0.01), and 51.9 (p < 0.01) at three-, six-, and nine-month time points, respectively (Figure 3A). The same held true for back pain intensity, which decreased from 7.5 to 6.0 (p < 0.01), 5.8 (p < 0.01), and 5.7 (p < 0.01; Figure 3B), as well as back pain frequency, which decreased from 7.8 to 6.4 (p < 0.01), 6.2 (p < 0.01), and 5.6 (p < 0.01; Figure 3C). VAS radiating right leg pain decreased insignificantly from 36.1 to 33.5, 27.5, and 28.9 (Figure 3A) while VAS radiating left leg pain decreased insignificantly from 32.7 to 31.4 and 24.4 at three and six months but decreased significantly to 20.3 at nine months (p < 0.05; Figure 3A). Leg pain intensity decreased insignificantly from 5.3 to 4.6 and 4.1 at three and six months but decreased significantly to 3.6 at nine months (p < 0.01; Figure 3B). Leg pain frequency decreased insignificantly from 5.0 to 4.7 and 4.2 at three and six months but decreased significantly to 3.1 at nine months (p < 0.01; Figure 3C). Daily function, as measured on the ODI, decreased significantly from 47.2 to 39.5 (p < 0.01), 38.1 (p < 0.01), and 39.4 (p < 0.01; Figure 3D).

**Figure 4:**
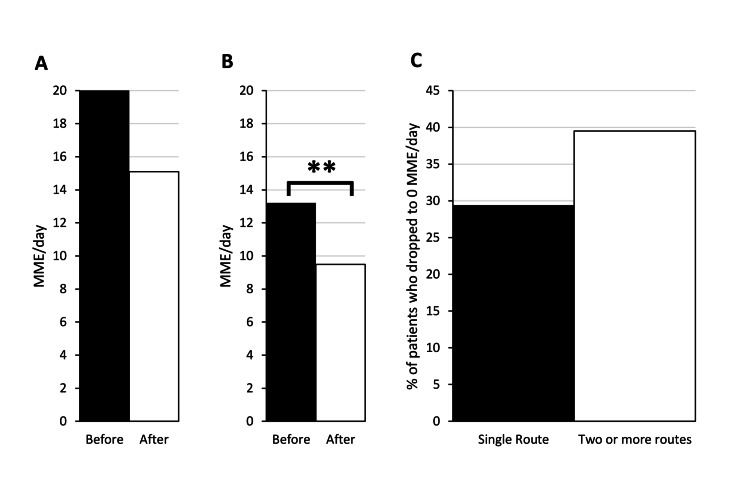
Patients on more than one route of administration show decreased opioid usage post-medical cannabis certification. Patients on a single route of administration (A) showed an insignificant drop in morphine milligram equivalents per day (MME/day) from 20.0 to 15.1 (n = 68, p = 0.055). Patients on two or more routes of administration (B) showed a drop from 13.2 to 9.5 (n = 75, **p < 0.01). Patients on a single route of administration and patients on two or more routes of administration had a 29.4% and 39.5% drop to zero rates, respectively (C).

Data on the route of MC administration were collected for 144 (77.0%) patients. A total of 68 patients (47.2%) used only a single route, 54 patients (37.5%) used two routes, 20 patients (13.9%) used three routes, and two patients (1.4%) used four routes. Vaporized oil and sublingual tincture were used most commonly, with 60 patients (41.7%) using each (Table 2). Among patients using only a single route of administration, vaporized oil and sublingual tincture were also used most commonly with 19 patients (27.5%) using vaporized oil and 16 patients (23.2%) using sublingual tincture (Table 3). The least commonly used route of administration was vaporized flower. For patients using a single route of administration, the average MME/day dropped insignificantly from 20.0 to 15.1 (n = 68, p = 0.054; Figure 4A). The percentage of those patients who dropped to 0 MME/day was 29.4% (Figure 4C). For patients using two or more routes of administration, the average MME/day dropped from 13.2 to 9.5 (n = 76, p < 0.01; Figure 4B). The percentage of those patients who dropped to 0 MME/day was 39.5% (Figure 4C).

**Table 2:** Number of patients using each medical cannabis route of administration.

Route of administration	N	%
Vaporized oil	60	41.7%
Sublingual tincture	60	41.7%
Topical	50	34.7%
Oral	43	29.9%
Vaporized flower	31	21.5%

**Table 3:** Number of single route users per medical cannabis route of administration.

Route of administration	N	%
Vaporized oil	19	27.9%
Sublingual tincture	16	23.5%
Topical	15	22.1%
Oral	10	14.7%
Vaporized flower	8	11.8%

Data on adverse effects were collected for 71 (38.2%) patients. Of respondents, 40 patients (56.3%) did not feel intoxicated or high, and 31 patients (43.7%) felt intoxicated or high (Table 4). Of the patients who felt intoxicated or high, 21 patients (29.6%) said that it did not interfere with their daily activities, two patients (2.8%) said that it made their day even better, and eight patients (11.3%) said that they did not like it or that it interfered with their daily activities (Table 4).

**Table 4:** Patient-reported adverse effects of medical cannabis.

Category	n	%
All respondents	71	100
I do not feel intoxicated/high	40	56.3
I feel intoxicated/high	31	43.7
It does not interfere with my daily activities	21	29.6
It makes my day even better (I enjoy it)	2	2.8
I do not like it (or it interferes with my daily activities)	8	11.3

## Discussion

In this study involving patients with chronic musculoskeletal noncancer back pain certified for MC, opioid prescriptions filled significantly decreased after MC certification. A significant decrease in opioids filled is also seen upon sub-analysis of the less than 15 MME/day group and the greater than 15 MME/day group. Moreover, patients in the less than 15 MME/day group had over a 35% chance of eliminating opioid use. These opioid reductions were also associated with improvements in pain scores and daily function. Our findings support the hypothesis that the introduction of MC for chronic low back pain is associated with a decrease in opioid prescriptions filled. Furthermore, we found that patients who use only a single route of administration do not show a significant decrease in opioid use while those using two or more routes do show a significant decrease, suggesting that multiple routes may be more effective in replacing opioids. Additionally, the majority of patients did not feel intoxicated or high from MC, and of those who did, only a small percentage of patients said it interfered with their daily activities. It should be noted that adverse effect results are limited given the variability in patient MC administration.

A 28.2% change in MME/day has been suggested as a minimal clinical important difference (MCID) [19]. Based on our data, MME/day dropped 27.0%, 39.4%, and 24.5% in all patients, patients below 15 MME/day, and patients above 15 MME/day, respectively. While patients below 15 MME/day would meet the criteria for MCID, they only saw an absolute change in 1.4 MME/day. Our data were presented as an average; however, individual patients did experience dramatic drops in MME/day that would be considered clinically significant. MCID in pain on the VAS is reported as 13 out of 100, and MCID in pain on numerical rating scales is reported as a change greater than two [14,15]. VAS back pain, as well as numeric back pain frequency, decreased by levels that would be considered clinically significant. MCID in the ODI scale has varying suggestions with the smallest threshold being a five-point change [16]. ODI saw a change in 9.1, which meets the smallest threshold. Despite the significant drop in ODI, patients still had a score of 39.5 suggesting that while MC may provide improvements in disability, MC is not sufficient on its own to completely alleviate disability associated with chronic low back pain.

Current literature is mixed over whether the introduction of MC is associated with decreased opioid use in patients with chronic pain. While some studies have shown decreased opioid usage with MC, other studies have reported no change or increased prescription drug use [10,20-26]. The majority of existing studies utilize patient surveys to measure opioid use [11,21-24]. Very few studies investigate how the use of MC affects opioid usage specifically for chronic musculoskeletal pain [10,27,28]. Our study adds to the current literature by providing an objective quantification metric to show that MC certification is associated with decreased opioid prescriptions filled in patients with chronic musculoskeletal back pain. To our knowledge, this is the only study that stratifies patients based on the level of opioid utilization. While our study does associate MC introduction with a decreased opioid prescription, other potential reasons for decreased opioid usage should be considered including a natural decrease in opioid usage or intentional weaning of opioids with concurrent MC prescription. These variables were not investigated in the present study.

The mechanism of action of MC involves two major components: THC and CBD. THC activates cannabinoid receptors type 1 and 2 (CB1 and CB2) [7]. THC activates these receptors to provide both analgesic and psychotropic effects. CBD, on the other hand, does not seem to act through CB1 or CB2 receptors but instead has agonistic activity on the serotonin 1A receptor (5-HT1A), adenosine A2A receptor, and the peroxisome proliferator-activated gamma receptor (PPAR-γ) [29]. As such, CBD provides analgesic properties through the anti-inflammatory pathways of those receptors without causing a psychotropic effect. The exact mechanism of action of analgesia provided by CBD requires further investigation.

Previous literature regarding the route of administration has shown that various routes differ in side effect profiles [30]. There is a scarcity of research comparing routes of administration in their effectiveness to control pain. To our knowledge, this is the first study to identify a number of routes as a potential predictor of treatment effectiveness.

While most cohort studies utilize a follow-up period within six months following MC initiation, a study conducted in California tracked long-term opioid usage of opioids for chronic back pain and showed that stopping opioid usage after MC initiation takes an average of six years [21,22,27]. Our study supports evidence that short-term opioid usage is diminished and potentially stopped within six months of MC certification but does not provide information on long-term therapy. Future analysis is required to corroborate those findings.

Currently, MC is recommended in a limited role to select patients with a terminal illness or chronic refractory pain due to an overall lack of substantial clinical evidence [31]. Our findings suggest that patients with low levels of baseline opioid use, specifically less than 15 MME/day, have a high chance of replacing opioids altogether with MC. Patients on high-dose opioids also see a significant decline in opioid usage. However, questions regarding the utility of MC are still warranted. Specifically, for patients below 15 MME/day, the risk of continued opioid usage is questionable. Studies have shown that for chronic pain, the duration of opioid therapy is more associated with the risk of overdose than the daily dose [32]. Still, the risk of the current opioid regimen must be weighed against the risk of MC introduction. Further study into the therapeutic efficacy and safety profile of MC is needed to make formal guideline recommendations.

Gaps in current literature include but are not limited to proper MC dosing, long-term efficacy, and side effect profiles. This study begins to look at which opioid usage profiles are best served with MC recommendations, but further study is needed to identify formal indications. Federal rescheduling of cannabis to a Schedule III drug like dronabinol, the FDA-approved synthetic form of THC, would significantly improve the ability to conduct these highly warranted studies.

This study has several limitations. First, it was an uncontrolled and observational study. Because MC remains a Schedule 1 drug, controlled trials are not currently possible without an “Investigational New Drug” trial approval from the US FDA. Tracking filled opioid prescriptions does not necessarily indicate the actual use of opioids. We could not account for any diversion of opioid usage. In addition, we could not track the acquisition of illicit opioids. Our study does not track long-term MC usage and may miss attrition. Due to the study design as well as a lack of data on MC safety profile, our study cannot directly comment on MC efficacy or current guidelines. This study did not allow for the correlation of pain and opioid reduction outcomes with specific cannabis products, phenotypes, cannabinoid ratios, or daily doses. Participants self-selected products at retail dispensaries and used a wide variety of cannabis and cannabis-infused products ad-libitum. Data on MC adverse effects have limited generalizability given the variability in patient MC administration.

## Conclusions

Among patients with chronic musculoskeletal noncancer back pain who were certified for MC, they filled a significantly reduced amount of opioid prescriptions post-MC compared to pre-MC. Upon MC certification, patients with lower levels of baseline opioid use have a high chance of stopping opioid use altogether. Patients show improved pain scores and daily function scores following MC certification. The use of multiple routes of administration simultaneously may be more efficacious in reducing opioid utilization.
